# Global proliferation of nonnative plants is a major driver of insect invasions

**DOI:** 10.1093/biosci/biae088

**Published:** 2024-10-18

**Authors:** Cleo Bertelsmeier, Aymeric Bonnamour, Eckehard G Brockerhoff, Petr Pyšek, Jiří Skuhrovec, David M Richardson, Andrew M Liebhold

**Affiliations:** Department of Ecology and Evolution at the University of Lausanne Switzerland; Department of Ecology and Evolution at the University of Lausanne Switzerland; Swiss Federal Research Institute WSL, Birmensdorf, Switzerland; Department of Ecology, Faculty of Science at Charles University, Prague, Czech Republic; Crop Research Institute, Prague, Czech Republic; Department of Ecology, Faculty of Science at Charles University, Prague, Czech Republic; Centre for Invasion Biology in the Department of Botany and Zoology at Stellenbosch University, Stellenbosch, South Africa; USDA Forest Service Northern Research Station, Morgantown, West Virginia, United States; Faculty of Forestry and Wood Sciences at the Czech University of Life Sciences Prague, Czech Republic

**Keywords:** human-mediated dispersal, facilitation, enemy release, introduction pathways, empty niche

## Abstract

Invasions by nonnative insect species can massively disrupt ecological processes, often leading to serious economic impacts. Previous work has identified propagule pressure as important driver of the trend of increasing numbers of insect invasions worldwide. In the present article, we propose an alternative hypothesis—that insect invasions are being driven by the proliferation of nonnative plants, which create niches for insect specialists and facilitate their establishment outside their native ranges where their hosts are planted or are invasive. We synthesize mechanisms by which plant invasions facilitate insect invasions, macroecological patterns supporting the tight link between plant and insect invasions, and case studies of plant invasions having facilitated subsequent insect establishment. This body of evidence indicates that plant invasions are a major driver of insect invasions. Consequently, the benefits of limiting the spread of nonnative plants include averting the proliferation of nonnative insects and their spillover onto native plant species.

Insects make up the majority of animal species, having colonized every major biome with the exception of most marine habitats. Insects also make up the majority of nonnative animal species and insect invasions are ubiquitous in all world regions (Seebens et al. 2017, Liebhold et al. 2018). Over the last 200 years, rates of insect invasions have increased worldwide (Roques et al. 2016, Bertelsmeier et al. 2017, Seebens et al. 2017), causing a wide range of ecological impacts, primarily through their feeding on plants but also by outcompeting native insect species, disrupting insect–plant mutualisms, altering pollination services and vectoring animal and plant diseases (Traveset and Richardson 2006, Kenis et al. 2009, Boyd et al. 2013, Hill et al. 2016). The socioeconomic consequences of these impacts on agriculture, human health and ecosystem services are manifold (Lounibos 2002, Aukema et al. 2010, Paini et al. 2016). However, despite their obvious importance as invaders, insects have received disproportionally less attention from invasion biologists than other taxonomic groups, especially plants (Pyšek et al. 2008, Edney-Browne et al. 2018). This may be because insects are typically small and only noticeable during part of the year. By contrast, plant invasions are often more visible, which draws more attention to them.

The number of insect species detected during import inspections vastly exceed that of nonnative species recorded as established (Turner et al. 2021), indicating that most insects transported to new regions fail to establish or have not yet established. To improve strategies for minimizing future insect invasions, it is crucial to develop a comprehensive understanding of the factors driving current invasion trends. Propagule pressure, associated with international trade and human travel, has been implicated as an important driver of insect invasions (Bertelsmeier et al. 2017, Brockerhoff and Liebhold 2017, Hulme 2021, Ollier and Bertelsmeier 2022, Fenn-Moltu et al. 2023, Liu et al. 2023). High propagule pressure can come from either many introduction events or many individuals being introduced in a single introduction event. The reasons that high propagule pressure might increase establishment success include increased genetic diversity and greater resistance to stochastic or density-dependent effects (e.g., Allee effects; Lockwood et al. 2005, Simberloff 2009). Most efforts to reduce problems associated with insect invasions focus on reducing propagule pressure (i.e., preventing the accidental transport of insects in trade and travel; Hulme 2013, Nahrung et al. 2023). However, we argue in the present article that considerable evidence points toward the establishment of nonnative plants as a major driver of the establishment of nonnative insect species—in particular, specialist herbivores (Liebhold et al. 2018, Bonnamour et al. 2023). Although propagule pressure is a necessary ingredient for any invasion, the increasing dominance of nonnative plants explains spatial variation in the number of historical insect invasions (Liebhold et al. 2018, Bonnamour et al. 2023) and therefore appears to be a major driver of insect invasions.

The establishment of nonnative plants may be a necessary precondition for the subsequent spread of nonnative insects because insect herbivores and plants have a long shared coevolutionary history that has spawned evolutionary radiations and parallel trends in diversification (Zeng et al. 2014). Because herbivores may, in turn, have coevolved specialist predators or parasites, an invasion by a plant can open up opportunities for establishment not only for herbivores directly using it as a resource but also subsequently by species of higher trophic levels such as parasitoids (figure 1; Weber et al. 2021). Nonnative plant species are spreading extensively in terrestrial and aquatic ecosystems around the world (Pyšek et al. 2020), facilitated by human-caused disturbance of natural ecosystems (Chytrý et al. 2008, Sánchez-Ortiz et al. 2020, Liu et al. 2023). When insects are transported to novel regions, they no longer face a landscape devoid of their preferred host plants but instead are reunited with hosts from their native range, which allows them to establish, thrive and spread (Gougherty and Davies 2022). Elevated diversity of nonnative plant species creates more ecological niches for arriving insect herbivores (Guo et al. 2019b, Ward et al. 2022), which, in turn, provides new niches for insect predators and parasitoids (figure 1; Wilson et al. 2013). In that way, plant invasions may have a catalytic effect on new invasions of diverse types of insects and the species relying on them. This concept of invasions by one or more species facilitating the invasion of another species has been termed *invasional meltdown* (Simberloff and von Holle 1999, Simberloff 2006, Braga et al. 2018).

**Figure 1.**
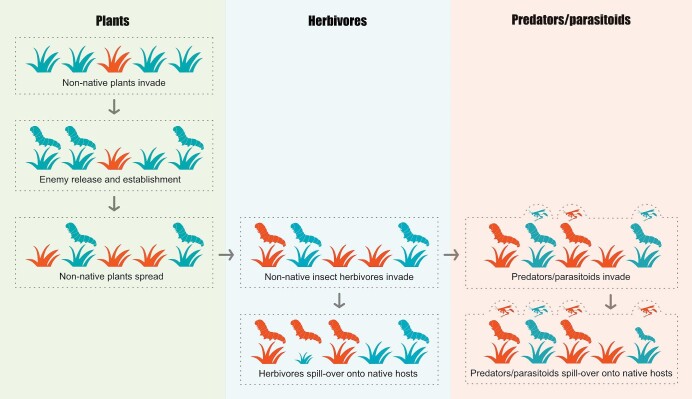
Mechanisms of nonnative plants facilitating the establishment of nonnative insect herbivores and higher trophic levels such as predators and parasites. Nonnative species are shown in red and native species in blue.

## Mechanisms: How plant invasions favor insect invasions

The exchange of plants became a truly global phenomenon since the end of the fifteenth century (di Castri 1989, van Kleunen et al. 2015). European explorers and plant hunters collected plants elsewhere and introduced them to Europe and also to newly settled areas to provide food for humans and animals and for medicinal and aesthetic purposes (di Castri 1989, Mack and Lonsdale 2001, Lenzner et al. 2022). Since then, nonnative plants have become increasingly dominant in the world's ecosystems (Stohlgren et al. 2011, Pyšek et al. 2017), following the establishment of self-sustaining populations that recruit from both escapes from cultivation and accidental introductions (Lambdon et al. 2008, Lehan et al. 2013, Saul et al. 2017). Today, more than 13,000 plant species are established outside of their native ranges (van Kleunen et al. 2015), leading globally to a decline in species diversity in plant communities (Pyšek et al. 2012) and strong biotic homogenization (Yang et al. 2021), a pattern that is also observed in insects (Aulus et al. 2024). The numbers of species that successfully naturalize in the new regions are still increasing (Seebens et al. 2017, Bonnamour et al. 2021) and will likely keep increasing in the future (Seebens et al. 2015).

Agriculture probably represents humanity's largest transformation of the world's flora, with 32% of the global land area devoted to agricultural use (Ellis et al. 2010); most agricultural crops are nonnative in most areas where they are planted (Young 2016) and they create ecological niches for insects that specialize on them (Paini et al. 2016). Likewise, forest plantations of nonnative tree species also provide new ecological niches for nonnative insect species in world regions where these insects could previously not exist (Wingfield et al. 2015). Planted forests (including plantations), many of which are using nonnative trees (Brockerhoff et al. 2008), now cover about 294 million hectares (ha), representing 7% of the world's total forest area (FAO 2020). But they are continuously expanding and likely to reach 20% by 2100 (Brockerhoff et al. 2013, Payn et al. 2015). For example, nonnative trees in the genera *Acacia, Eucalyptus* and *Pinus* are extensively planted in forestry operations throughout much of the southern hemisphere. Plantations of nonnative *Pinus radiata* in New Zealand, Australia, South Africa, and Chile exceed 4 million ha (Brockerhoff et al. 2023), and nonnative plantations of *Eucalyptus* species cover more than 20 million ha, with the largest area in China, Brazil, and India (Wingfield et al. 2015). Australian *Acacia* species have been planted in many parts of the world to support commercial forestry and for many other purposes (Richardson et al. 2023). In most parts of the world, widespread plantings and invasions of *Acacia* species are more recent than those of eucalypts and pines; insect invasions and tree-health problems associated with nonnative *Acacia* species are therefore relatively few compared with those in eucalypts and pines (Hurley et al. 2023). Nonnative plants in arboreta and urban settings also provide ecological niches that facilitate invasions of insects from the native ranges of these plants. During the eighteenth and nineteenth centuries, collecting live “exotic” plants became very popular, and important botanical institutions were created, such as the Royal Botanical Gardens, Kew, in the United Kingdom, and the Leiden Hortus Botanicus, in the Netherlands (Mack and Lonsdale 2001, van Kleunen et al. 2018). More than 160,000 vascular plant species are now grown in botanical gardens around the world, representing almost 50% of the global known vascular flora (van Kleunen et al. 2018). Some of the species in botanic gardens are native where they are grown, but a large fraction is nonnative. Therefore, arboreta provide a virtual smorgasbord of host plants for arriving nonnative insect species and there are several records of insects and plant pathogens that have established there (Wondafrash et al. 2021).

In addition, a large fraction of plantings in urban landscapes is dominated by nonnative plants. For example, in UK gardens, about 70% of plants are nonnative (Loram et al. 2008). Urban areas are also well connected to introduction pathways as most imports arrive in urban areas. So, the combination of high arrival rates and facilitation of establishment by copious numbers of nonnative plants leads to urban areas being the site of most insect invasions (Branco et al. 2019, Ward et al. 2019). It has been argued that urban plantings are the first places where for nonnative insect species can be detected because they are often the first point of contact for arriving species (Branco et al. 2015, Paap et al. 2017). However, additional populations of the host plant may be located at great distances from those arrival points. Therefore, regional transport routes are a key factor determining further spread of nonnative insects (Gippet et al. 2019). Indeed, many invasive plant species are now so well established and cover large areas that they can provide stepping stones for a newly arriving insect species that can move rapidly over great distances. For example, the nonnative pine bark beetle, *Hylurgus ligniperda* has invaded much of the southern hemisphere (i.e., Australia, New Zealand, South Africa, and southern South America) and spread quickly, using planted pines as stepping stones (Brockerhoff et al. 2006, Faccoli et al. 2020). Overall, nonnative trees are widespread across urban areas and are likely to provide increased host availability compared with surrounding forests (e.g., Augustinus et al. 2024).

Even protected areas, including those located at great distances from human-dominated ecosystems and seemingly isolated from the major pathways of plant introductions, are increasingly affected by nonnative plant invasions (Foxcroft et al. 2013). So far, however, less than 3% of protected areas are invaded by nonnative invertebrates, perhaps because the low levels of human activity provide few opportunities for introduction (Liu et al. 2020). Alternatively, there may be substantial time lags between the establishment of plants in these areas and the subsequent spread of insects.

### Nonnative insects are continuously arriving

Nonnative insects are continuously moving over long distances, mainly through human transport but, in some cases, also via natural dispersal. Some insect species are able to perform natural long-distance dispersal flights and can thereby spread to new regions and across physical barriers (Chapman et al. 2015). Extreme climatic events such as storms and hurricanes also can cause long-distance dispersal of insects. For instance, there is evidence that several species of insects have dispersed from Australia to New Zealand across open ocean by suitable air currents (Close et al. 1978). Although it is likely that many different species of insect have been translocated between continents over the last centuries via wind, birds or marine rafting (Holzapfel and Harrell 1968), most such translocations have not resulted in new invasions because of a lack of hosts. Insects are now mainly introduced through international trade and transport (Gippet et al. 2019). In particular, the worldwide movement of plants can directly promote insect invasions by providing pathways by which insects can travel long distances. The global trade of live plants is well known as the historically dominant pathway for insect introductions (Kenis et al. 2007, Smith et al. 2007, Liebhold et al. 2012, Meurisse et al. 2019). These natural and human-meditated long-distance dispersal events occur continuously, but many of them do not lead to insect establishment, probably most importantly because suitable host plants are absent or scarce in the introduced range. Indeed, host plant availability is a major factor determining the distribution and spread of nonnative insects (Zalucki and Clarke 2004, Dang et al. 2021). This will however depend on the host specificity of individual insect species; the availability of hosts from the native range will be more significant for specialists, whereas the establishment of generalists is less likely to be facilitated by nonnative plants.

### Filling the vacuum of insect diversity on introduced host plants

In their native ranges, most plants are associated with a large number of more or less specialized herbivorous and other insects (Strong et al. 1984), while they are in their nonnative ranges, they are often, at least initially, exploited by fewer insects recruited from the native insect fauna (Goßner et al. 2009, Pyšek et al. 2011, Branco et al. 2015). This leads to enemy release, especially when there are no closely related (e.g., congeneric) native plants in the invaded region (Procheş et al. 2008, Carrillo-Gavilán et al. 2012, Branco et al. 2015). This is because most herbivorous insect species are specialists (Forister et al. 2015). However, nonnative plants offer niches that facilitate the establishment of insects outside their native ranges where their hosts are planted and, over time, specialists from the native range can catch up with their host plants (figure 1; Hawkes 2007, Brockerhoff and Liebhold 2017). When no closely related native plants are present, accumulating specific natural enemies in the new range takes time; it has been shown that for pathogens, this process happens on a scale of centuries (Mitchell et al. 2010). Similarly, nonnative plants have been shown to benefit from release from herbivore enemies most during early invasion stages, but this release is diminished 50–200 years after their invasion (Hawkes 2007). The diminished herbivore richness may result in high rates of growth and reproduction for nonnative plants that substantially exceed rates occurring in their native range through enemy release (Keane and Crawley 2002). Although the universality of this process in nonnative plants has been questioned (Schultheis et al. 2015, Brandenburger et al. 2020), recent analyses indicate its widespread and common occurrence (Xu et al. 2021). The spread of nonnative plants leads initially to a “vacuum” of insect diversity (specialist herbivores that are present in the native but absent in the introduced range). Over time, this vacuum is filled as specialist herbivores feeding on these nonnative plants can establish, which, in turn, results in a diminution of the benefits of enemy release with time since the introduction of the host plant (Hawkes 2007, Chen 2016).

For example, early *Eucalyptus* plantations benefited from a largely herbivore-free environment in their nonnative ranges, but the rate of spread of nonnative pests of *Eucalyptus* has increased nearly fivefold since the 1980s (Hurley et al. 2016). Similarly, the spread of black locust (*Robinia pseudoacacia*), a tree species that has invaded every temperate continent, has led to a parallel, but delayed, spread of insect herbivores that feed on it (Medzihorský et al. 2023). The reunification of nonnative plants with herbivore species accidentally introduced from their native ranges can result in a pronounced decrease in the benefits of enemy release experienced by these plants. Shaw and colleagues (2018) show that prior to 2010, all introductions of natural enemies affecting weeds in the European Union have been unintentional, but several of these species have substantially reduced the abundance of their hosts.

Once they have established, nonnative herbivores are sometimes able to expand their host range beyond their nonnative host to include native plant communities, sometimes with detrimental consequences to those communities (White and Whitham 2000, Gougherty and Davies 2021). This spillover effect may occur when insects reach high densities on their preferred hosts and then begin exploiting less preferred neighboring plants (White and Whitham 2000), or they simply may be able to feed on native plants that are phylogenetically related to their nonnative hosts (Gougherty and Davies 2021). For instance, the North American chrysanthemum lace bug (*Corythucha marmorata*), which was unintentionally introduced to Japan, was shown to feed on native Asteraceae that grow next to its preferred nonnative host (*Solidago altissima*; Sakata and Craig 2021) but also on less related plants in the Convolvulaceae and Solanaceae families (Rizkawati et al. 2023). In another example, the invasion of North America by the spotted lanternfly (*Lycorma delicatula*) has been facilitated by the ubiquity of its preferred host, the nonnative tree of heaven (*Ailanthus altissima*), but once established, this sap-feeding insect of moderate host specificity can also feed on and damage native trees such as black walnut (*Juglans nigra*) and cultivated crop plants such as grape (*Vitis vinifera*; Murman et al. 2020). The spillover may occur after some initial time lag following the establishment of the nonnative insect. However, typical time lags are so far unknown.

Nonnative plants can also facilitate invasions of nonherbivorous insects, such as pollinators and ants. Nonnative pollinators tend to visit more nonnative than indigenous plants, suggesting that nonnative plants might act as stepping stones facilitating pollinator invasions (Morales and Aizen 2002, Fontúrbel et al. 2023). Furthermore, the widespread presence of nonnative plants can facilitate invasions of specialist pollinators. For example, the cultivation of squash (*Cucurbita* spp.) throughout North America has enabled the invasion of the pollen specialist *Peponapis pruinosa* from the native range of squash in southern Mexico (López-Uribe et al. 2016). Nonnative herbivorous insects can also facilitate the spread of other insects that have mutualistic relationships with them. For instance, the abundance of the red imported fire ant, *Solenopsis invicta*, in North America increases with the abundance of the nonnative honeydew-producing mealybug *Antonina graminis* (Helms et al. 2011). The abundance of this mealybug is positively affected by the abundance of the nonnative host grass *Cynodon dactylon*. Therefore, the abundance of the nonnative plant indirectly facilitates the fire ant invasion.

A substantial fraction of insect species worldwide are predators and parasitoids, although the true richness of parasitoids, especially in the understudied Hymenoptera, is probably at least twice the number of described species (e.g., Dolphin and Quicke 2001). Similar to the way plant invasions can create niches for herbivorous insects and facilitate their invasion, invasions of herbivorous insects create niches for insects at higher trophic levels (predators and parasitoids) and thereby facilitate their invasions. The unintentional introduction of parasitoids and predators of insect pests is a phenomenon recently referred to as “accidental biocontrol,” and several studies show that this phenomenon is common worldwide (Weber et al. 2017, 2021). It is estimated that 35% of parasitic Hymenoptera in the United States and 32% of arthropod predators and parasitoids in Europe were introduced accidentally (Weber et al. 2017). Similarly, Charles (1998) reported that 79% of parasitoids attacking nonnative fruit crop pests in New Zealand arrived accidentally. In most cases, it remains unknown if these species entered with their insect hosts or were hitchhikers in trade and human travel. However, it is clear that for many species, establishment would not have been possible without the presence of hosts from their native range. In the Mediterranean region, several specific parasitoids of nonnative eucalypt pests are thought to have been accidentally introduced after their host (Kenis et al. 2017). For example, the parasitoid *Psyllaephagus bliteus* was first observed in Portugal in 2011, 4 years after the first record of its nonnative host, *Glycaspis brimblecombei*, that feeds on eucalypts (Boavida et al. 2016).

Much like invasions of insect herbivores, accidental invasions of parasitoids facilitated by invasions of their hosts can spill over onto other hosts, adversely affecting native fauna (Mason et al. 2017). Teulon and colleagues (2008) note that a very small number (approximately 15) of aphids are native to New Zealand and these are mostly rare; however, they are being adversely affected by accidentally introduced aphid parasitoids. More than 110 species of nonnative aphids have invaded New Zealand (Brockerhoff and Liebhold 2017) and, apparently, have facilitated invasions by at least 10 species of nonnative aphid parasitoids, some of which have been found to spillover onto native aphid hosts (Teulon et al. 2008). Together, these pieces of evidence suggest that the establishment of host plants is a crucial prerequisite to the subsequent establishment of insects.

## Macroecological patterns: Empirical evidence for the link between plant and insect invasions

Analysis of geographical variation in the numbers of naturalized or invasive species can be used to identify dominant drivers of invasions (Pyšek et al. 2010, Dawson et al. 2017, Essl et al. 2019). In a global study, Liebhold and colleagues (2018) analyzed variation in nonnative insect richness among 44 land areas, ranging from small oceanic islands to entire continents. Using structural equation modelling, they found that the most important determinants of nonnative insect richness are native and nonnative plant richness. Several studies report that variables related to human population size and economic activity explain variation in numbers of naturalized plant species, presumably because they are correlates of plant propagule pressure (Pyšek et al. 2010, Wohlwend et al. 2021). Similar measures of human activity have also been reported to explain variation in nonnative insect species numbers (Lantschner et al. 2020, Trombik et al. 2023), but these correlations may arise either directly as a result of associations with propagule pressure or indirectly as a result of their impacts on plant invasions that subsequently facilitate insect invasions.

Recent studies indicate that there may be a substantial temporal lag in the link between plant invasions and insect invasions. Bonnamour and colleagues (2023) analyzed the association between current insect invasions and historic plant invasions among biogeographic regions; they found that recent detections of insect invasions (i.e., prior to 2010) are well explained by cumulative plant invasions prior to 1900. In fact, these historical plant invasions were a better predictor of current insect invasions than more recent plant invasions (Bonnamour et al. 2023). Global flows of invasive plants also explained much more of the variation in global flows of invasive insect than did trade between regions. The long time lag between plant and insect invasions is probably attributable to the combined effects of several processes. First, following initial plant naturalization, it takes time for a given species to spread and become abundant within the region where it first arrived (e.g., Gassó et al. 2010). Second, the reporting lag for insect invasions may be quite long; for example, Maclachlan and colleagues (2021) estimated an approximately 80-year median lag between the establishment and discovery of invasions by Hemiptera in the United States. One implication of the long lag between plant invasions and discoveries of insect invasions is that there is likely a relatively large invasion debt for insects worldwide. This means that there may already be numerous insects at early stages of establishment, but they might still be so rare and cryptic that many of them will not be detected for many years or decades.

Potentially, plant diversity can have both positive and negative effects on the population growth and invasion success of herbivorous insects. According to the resource concentration hypothesis or facilitation effect, higher densities of host plants facilitate population growth by minimizing dispersal loss (Root 1973, Stephens and Myers 2012). In a similar fashion, the presence of large numbers of nonhost plants may depress insect herbivore population growth, a phenomenon termed *the dilution effect* (Jactel and Brockerhoff 2007). Guo and colleagues (2019a) analyzed variation in numbers of tree-feeding insect pests per county across the United States and found a hump-shaped relationship between nonnative forest insect species richness and tree species richness. On further dissection of this relationship, they determined that it likely arose from the combination of a positive effect of host tree richness on nonnative insect richness (as a result of the facilitation effect) and a negative effect of nonhost richness (as a result of the dilution effect). In a subsequent analysis of the same data, Ward and colleagues (2022) found evidence of a host tree facilitation effect on the establishment of some nonnative tree-feeding insects (especially sap-feeding insects), a dilution effect by which the presence of nonhosts inhibited establishment of other insect species and several species that did not exhibit either a facilitation effect or dilution effect. However, taken together, these studies provide evidence that the diversity of plants, both nonnative and native, can promote insect invasions.

A common observation in macroecological analyses of invasions is an association of native and nonnative species richness. This pattern is opposite the expectation of the biotic resistance hypothesis, originally posited by Elton (1958), which predicts that systems with high species diversity are more resistant to invasion because of a higher proportion of niches already being filled. In plants, the evidence is mixed: In some cases, nonnative plant richness is negatively related to native plant diversity (Tilman 1997, Naeem et al. 2000), but in other studies, it is a positive relationship (Stohlgren et al. 2003, Pyšek et al. 2017). This discrepancy is mostly because of the spatial scale of observation and can be explained by covarying external factors; at the large scale, the same abiotic conditions that promote a high diversity of native species (climate, substrate, habitat heterogeneity, etc.) also support a high diversity of nonnative floras (Shea and Chesson 2002). Fridley and colleagues (2007) termed this phenomenon the *invasion paradox*, because the biotic resistance is mostly observed at small spatial scales but is reversed at larger scales (see also Rossignaud et al. 2022). The association between native and nonnative species richness has also been explored in insects at a macroecological scale. In a study of geographical variation in arthropod richness across the Azorean archipelago, Borges and colleagues (2006) found that nonnative arthropod species richness was strongly correlated with native arthropod species richness. A similar correlation was found in the global macroecological study on insects by Liebhold and colleagues (2018). These positive correlations between the numbers of native and nonnative insect species may result from there being generally more ecological niches in some regions that support more native and nonnative insect species. Alternatively, in some regions, there may be factors (e.g., climate, soils, land area) that promote plant diversity (native and nonnative), and this, in turn, promotes both native and nonnative insect diversity, thereby causing the correlation. Overall, these macroecological studies have revealed evidence that temporal dynamics and spatial patterns of plant and insect invasions are tightly linked.

## Case studies

Many widely abundant nonnative plant species have associated specialist insects that invaded from the nonnative plant's native range and were reunited with the plant in its nonnative range. Surprisingly, however, research that tests explicitly whether the establishment of a certain host plant was a necessary precondition for the establishment of a specific insect species and subsequent spillover to native plant species is scarce. In the present article, we will focus on well described examples that illustrate the fundamental role that plant invasions play in facilitating insect invasions (table 1).

**Table 1.** Examples of insect herbivore species whose invasion was facilitated by plants from the same native range.

Nonnative plant	Origin	Insect	Invaded range	Spillover onto native hosts?	Likely pathway	References
*Ailanthus altissima* (tree of heaven)	East Asia	*Lycorma delicatula* (spotted lanternfly)	North America	Yes, *Vitis, Acer, Malus, Populus, Salix*	Plants	Urban and Leach 2023
*Ailanthus altissima* (tree of heaven)	East Asia	*Samia cynthia* (ailanthus silkmoth)	North America, South America, Europe, Australasia	Yes, *Ricinus* (castor bean) and other plants	Intentional introduction	Baker 1985, Frank 1986
*Asclepias* spp.	North America	*Danaus plexippus* (monarch butterfly)	Oceania, Australasia, Europe	No	Wind dispersal, hitchhiking	Zalucki and Clarke 2004, Pierce et al. 2014
*Cucurbita pepo* (squash)	Central and Southern Mexico	*Peponapis pruinosa* (squash bee)	Conterminous United States	No	Wind	López-Uribe et al. 2016
*Eucalyptus* spp.	Australasia	43 species	Europe, North America, Africa, South America	No	multiple	Hurley et al. 2016
*Gossypium* spp. (cotton)	Neotropics	*Anthonomus grandis* (boll weevil)	Conterminous United States, South America	Yes, *Anthonomus grandis* (boll weevil)	Wind	Kim and Sappington 2006, Sánchez-Reyes et al. 2022
*Picea abies* (Norway spruce)	Europe	*Ips typographus, Dendroctonus micans*	Continental Europe and United Kingdom	No	Wood	Mayer et al. 2015
*Pinus radiata*, other *Pinus* spp.	Europe and North America	Numerous species of insects	Southern hemisphere	No	Wood, plants	Brockerhoff et al. 2023
*Pueraria* spp. (kudzu)	East Asia	*Megacopta cribraria* (kudzu bug)	North America, Indomalaya	Yes, *Glycine max* (soybean) and other species	Plants	Ruberson et al. 2013
*Robinia pseudoacacia* (black locust)	Eastern North America	8 species	Europe, North America, Australasia	Yes	Plants	Medzihorský et al. 2023
Diverse trees and shrubs	South America, Europe, Asia, Australia, Indomalaya	46 species of Psylloidea (Hemiptera)	North America	Maybe	Wood, plants	Horton et al. 2021

Tree of heaven, *A. altissima*, is native to east Asia but is one of the most widespread nonnative woody plants in virtually every part of the temperate world. This species spreads very fast, easily colonizes disturbed areas, and exhibits remarkable growth (Kowarik and Säumel 2007). Over time, several herbivores associated with it in its native range are catching up and invading portions of its invaded range (Ding et al. 2006). Examples include the spotted lanternfly (*L. delicatula*), the brown marmorated stink bug (*Halyomorpha halys*), and the ailanthus silk moth (*Samia cynthia*). The ailanthus silk moth was introduced to several world regions for purposes of silk production but escaped cultivation and continued to spread via natural dispersal into areas where *A. altissima* is abundant (Frank 1986). Both the spotted lanternfly and the brown marmorated stinkbug most likely invaded new regions as hitchhikers in cargo and are considered nuisance pests because they frequently reach very high densities near human settlements (Leskey and Nielsen 2018, Urban and Leach 2023). Furthermore, although both species prefer *A. altissima* as a host, both also feed opportunistically on economically important forest and crop plants (e.g., *Acer, Prunus, Vitis*) causing economic impacts in those sectors, providing an example of the spillover effect. Finally, *H. halys* has facilitated the invasion of at least one insect parasitoid species, *Trissolcus japonicus*. Starting in 2007, this species was being considered for release as a biological control agent targeting *H. halys* populations in North America, but in 2014, it was discovered that the parasitoid species had already invaded accidentally (Talamas et al. 2015). Nonnative populations were also recently discovered in Europe, having also arrived accidentally and established, apparently because of the widespread abundance of its host *H. halys* (Stahl et al. 2019).

Another example of an insect invasion that has been facilitated by the invasion of its host is provided by the monarch butterfly, *Danaus plexippus*. In its native North American range, it is an iconic species known for its annual long-range migrations to and from overwintering locations. However, over the last 100 years, the species has established nonnative populations across the Caribbean, Pacific islands, Australasia, Atlantic islands, and the southern Iberian Peninsula (Nail et al. 2019). Zalucki and Clarke (2004) speculate that invading populations arrived through some combination of hitchhiking with cargo and windborne dispersal. However, in these nonnative regions, its hosts (milkweeds in the subfamily Asclepiadoideae) are not native but are widely abundant as nonnative weeds or ornamental plants. Therefore, the worldwide spread of Asclepiadoideae through introductions, naturalization and invasion has paved the way for the global spread of monarchs.

The genus *Pinus* (pines) is one of the most abundant and diverse woody plant genera in the northern hemisphere. Although pines are not native to the southern hemisphere (except *Pinus merkusii*, which just crosses the equator in Indonesia), they are widespread and highly abundant there because of large-scale plantings for production forestry (Sedjo 1999) and because of the invasiveness many pine species exhibit in certain habitats (Richardson et al. 1994). The success of both planted and invasive pines can be attributed, in part, to their escape from their natural antagonists, mainly insects and plant pathogens, that are present in their native ranges and may limit their growth and reproduction (Richardson et al. 2007). However, numerous species of insect herbivores and plant pathogens that use pines as their main or only host have been accidentally transported to the southern hemisphere where, over time, many have found host pines and successfully established (Burgess and Wingfield 2001, Wingfield et al. 2006, Brockerhoff et al. 2023). The first prominent pine insect to invade the southern hemisphere was the European woodwasp, *Sirex noctilio*. Establishments were detected first in New Zealand around 1900 and subsequently in all southern hemisphere regions with pine plantations: Australia, South Africa, and South America (Slippers et al. 2015), where it is often considered one of the most important pests of pines. Other well-known illustrative cases include the North American pine bark beetle *Ips grandicollis* and the European pine shoot moth, *Rhyacionia buoliana*, which invaded Australia and Chile, respectively (Neumann 1987, Toro and Gessel 1999). All these invasions can be attributed mainly to the widespread presence of pines, their main or only host plants. Interestingly, these and many other pine insects are far more abundant and much more damaging in the invaded southern hemisphere regions than in their native ranges (where they are considered minor pests). This can be attributed to the combination of highly susceptible plants and the lack of specialized natural enemies of these insects. Many other potentially damaging insect species in the native range of pines have not yet invaded the southern hemisphere but hold potential for invasion, potentially diminishing the benefits of enemy release currently experienced by nonnative pines and decreasing the productivity of pines in forestry (Lantschner et al. 2017, Brockerhoff et al. 2023).

A final example is provided by potatoes (*Solanum tuberosum*), which are one of the globally most important staple foods and are grown on all continents (Singh and Kaur 2016). The ancestors of the main potato varieties originate from western South America, but other related wild potato species occur as far north as the southern United States (Ames and Spooner 2008, Singh and Kaur 2016). From the sixteenth century, potatoes were introduced to Europe and, subsequently, most of the rest of the world (Ames and Spooner 2008, Sauer 2017). The Colorado potato beetle (CPB), *Leptinotarsa decemlineata*, is considered the most important defoliator of potatoes and one of the most important potato pests in the northern hemisphere; infestations can lead to complete defoliation and crop loss (Hare 1990). CPB is endemic to North America, and the pest populations of CPB originate from the southern Plains of the United States (Hare 1990, Izzo et al. 2018). There, it made a host shift from native Solanaceae to cultivated potatoes around the mid-1800s (Hare 1990). Despite considerable efforts to prevent its introduction to Europe, it became established in the early 1900s and spread quickly through much of Europe and all the way to northeast Asia. Because potatoes are the main host of pest populations of CPB, the large-scale cultivation of potatoes in their nonnative range in Europe and Asia clearly facilitated its invasions, and the cultivation of potatoes in North America enabled the expansion of CPB's host range to potatoes in the first place.

## Conclusions

The evidence compiled in the present article supports our hypothesis that plant invasions are a crucial determinant of insect invasions. The close associations that many plants and insects form have evolved over millions of years. Consequently, the availability of host plants is a fundamental factor limiting the establishment success of nonnative insects. Plant invasions facilitate insect invasions directly by providing ecological niches for arriving insect herbivores, and indirectly by favoring the establishment of insect predators and parasitoids. Macroecological analyses support the hypothesis that nonnative plant richness is a major determinant of nonnative insect richness. Global flows of historical plant invasions are closely associated with flows of insect invasions a century later (Bonnamour et al. 2023); the existence of this century-long lag can be explained by the time required for nonnative plants to become widespread before functioning as stepping stones for subsequent insect invasions. Although there is macroecological evidence for such broad associations between nonnative plants and insects, more research is needed to document the invasions of specific pairs of plants and insects more generally and to test for time lags between the establishment of the host plant and the insect species and subsequent spillover to native plant species.

Overall, our synthesis provides evidence for mechanisms and global patterns supporting the links between plant and insect invasions. Variation in numbers of insect invasions worldwide are much more closely related to variation in numbers of plant invasions than they are to proxies of propagule pressure (Liebhold et al. 2018, Bonnamour et al. 2023). Although propagule pressure is a necessary ingredient of any invasion, rates of insect invasions are instead much more strongly limited by the availability of plants. Current biosecurity practices mainly focus on prevention of new arrivals of insects, but limiting the accidental spread of nonnative plants is also important for limiting insect invasions in the future. In this way, controlling the spread of undesired nonnative plant species would not only be beneficial because it mitigates the impacts of the plant species themselves, it would also reduce spillover of associated nonnative insects to native plant species. Although nonnative crops may host nonnative insects, limiting their spread is not an option because of the benefits that they provide to humanity.

## Author Biography

Cleo Bertelsmeier (cleo.bertelsmeier@unil.ch) and Aymeric Bonnamour are affiliated with the Department of Ecology and Evolution at the University of Lausanne Switzerland. Eckehard G. Brockerhoff is affiliated with the Swiss Federal Research Institute WSL, in Birmensdorf, Switzerland. Petr Pyšek is affiliated with the Department of Ecology, in the Faculty of Science at Charles University, in Prague, in the Czech Republic. Jiří Skuhrovec is affiliated with the Crop Research Institute, in Prague, in the Czech Republic. David M. Richardson is affiliated with the Department of Ecology, in the Faculty of Science at Charles University, in Prague, in the Czech Republic, and with the Centre for Invasion Biology in the Department of Botany and Zoology at Stellenbosch University, in Stellenbosch, South Africa. Andrew M. Liebhold is affiliated with the USDA Forest Service Northern Research Station, in Morgantown, West Virginia, in the United States, and with the Faculty of Forestry and Wood Sciences at the Czech University of Life Sciences Prague, in the Czech Republic.
